# Contractile activity is required for Z-disc sarcomere maturation *in vivo*

**DOI:** 10.1002/dvg.22851

**Published:** 2015-05-12

**Authors:** Timothy J Geach, Elizabeth MA Hirst, Lyle B Zimmerman

**Affiliations:** 1 Division of Developmental Biology, MRC National Institute for Medical Research, The RidgewayMill Hill, London, United Kingdom

**Keywords:** sarcomere, Z-disc, muscle, Xenopus, contractility

## Abstract

Sarcomere structure underpins structural integrity, signaling, and force transmission in the muscle. In embryos of the frog *Xenopus tropicalis*, muscle contraction begins even while sarcomerogenesis is ongoing. To determine whether contractile activity plays a role in sarcomere formation *in vivo*, chemical tools were used to block acto-myosin contraction in embryos of the frog *X. tropicalis*, and Z-disc assembly was characterized in the paralyzed *dicky ticker* mutant. Confocal and ultrastructure analysis of paralyzed embryos showed delayed Z-disc formation and defects in thick filament organization. These results suggest a previously undescribed role for contractility in sarcomere maturation *in vivo*. genesis 53:299–307, 2015. © 2015 The Authors. Genesis Published by Wiley Periodicals, Inc.

## INTRODUCTION

Sarcomeres are the basic myofibril unit fundamental for muscle contraction. Each myofibril consists of long F-actin filaments cross-linked at 2-μm intervals by multiprotein complexes called Z-discs, which define the boundary of a single sarcomere. Myosin-containing thick filament bundles (A-bands) occupy the space between each Z-disc, and interdigitate with F-actin in response to ATP to facilitate muscular contraction (Ehler and Gautel, 2008; Sparrow and Schock, 2009). Z-discs serve three main functions in the sarcomere: (1) stabilizing F-actin filament structures, (2) allowing force transfer between individual sarcomeres, and (3) acting as signaling centers communicating with the nucleus (Clark *et al*., 2002; Knoll *et al*., 2011; Sanger and Sanger, 2008). Z-discs themselves are linked to the surrounding sarcolemma by focal adhesion-like complexes called costameres (Samarel, 2005).

Although mature myofibril structures are well described, sarcomere formation *in vivo* remains poorly understood. Holtzer *et al*. (1997) proposed that thick filaments and Z-discs assemble independently and are stitched together at the end of an elongating myofibril. Another model suggests that the giant structural protein titin, which, in mature sarcomeres, is anchored in the Z-disc and extends to the M-band, forms a scaffold on which all other components assemble (Ehler *et al*., 1999). It has been proposed that Z-bodies, aggregations of Z-disc-specific proteins such as α-actinin, are organized on F-actin filaments by non-muscle myosin II (NMMII) forming “premyofibrils.” Non-muscle myosin is then replaced by sarcomeric myosin, and Z-bodies fuse to form Z-discs as the sarcomere matures (Dabiri *et al*., 1997; Du *et al*., 2003; Sanger *et al*., 2005). Finally, in an extension to the premyofibril model, integrins may also play a role in lining up Z-discs by acting as initial focal points for Z-disc attachment to the sarcolemma (Sparrow and Schock, 2009). Although contractile activity is the primary function of the mature sarcomere, the role of contraction in sarcomerogenesis has remained understudied.

Studies dating back to the early 1980s questioned the influence of innervation on specification and development of muscle. Surgical excision of the neural tube from chick embryos led to reduced growth in brachial muscle (Butler *et al*., 1982), and paralysis with *d*-tubocurarine inhibited formation of secondary myotubes (McLennan, 1983). In both studies, no change in the initial formation of muscle cells was observed, suggesting that neural stimulation is required for muscle maturation and growth but not initial specification. Harris (1981) found that tetrodotoxin -paralyzed muscles were smaller and had regions with few myotubes, despite relatively normal distribution of acetylcholine receptors. Conversely, blocking contraction of cultured rat myocytes with tetrodotoxin prevented formation of Z-discs and A-bands, which recovered upon removal of the agent (De Deyne, 2000), whereas inhibiting myosin ATPase activity (Kagawa *et al*., 2006; Ramachandran *et al*., 2003; Soeno *et al*., 1999) or calcium signaling (Ferrari *et al*., [Bibr b14],[Bibr b13]) produced thinner, branched myofibrils without affecting sarcomere number. Similarly, treatment of neonatal cultured myocytes with the myosin II inhibitor blebbistatin disrupted sarcomere formation (Skwarek-Maruszewska *et al*., 2009). Although innervation is clearly required for maturation and growth of muscle, the role of contractile activity in initial stages of sarcomerogenesis remains murky.

*Xenopus* sp. are excellent models in which to study sarcomerogenesis *in vivo*. Most skeletal muscle develops from mesodermally derived somites flanking the neural tube and notochord. As the embryo lengthens, somites rhythmically bud from the presomitic mesoderm. This process, known as somitogenesis, is tightly regulated by waves of gene expression, including members of the *wnt*, *fgf*, and *notch*/*delta* pathways (reviewed in Pourquie, 2011). In amphibians, the somites contribute to a transparent tail readily imaged by confocal microscopy. The diploid species *Xenopus tropicalis* is a highly flexible vertebrate model in which forward and reverse genetics (Goda *et al*., 2006; Young *et al*., 2011) can be combined with small molecule studies (Wheeler and Liu, 2012), genomic resources (Gilchrist *et al*., 2004; Hellsten et al., 2010), and conventional embryological and gain-of-function molecular techniques. We have identified a number of mutations affecting muscle development and/or sarcomere formation (Abu-Daya *et al*., 2009; Geach and Zimmerman, 2010; Goda *et al*., 2006), which provide a valuable resource for the study of myofibrillogenesis.

We previously described the *X. tropicalis* mutant *dicky ticker* (*dit*), in which thick filaments fail to form due to a mutation in the myosin co-chaperone *unc45b* (Geach and Zimmerman, 2010). We also noted that Z-disc assembly was significantly delayed in *dit* mutants. Based on the failure of α-actinin precursors to polymerize before thick filaments form in *unc45b* mutants as well as the timing of myosin chaperone expression (Etard *et al*., 2007; Geach and Zimmerman, 2010), Myhre and Pilgrim (2012) recently hypothesized that *unc45b* could directly regulate Z-disc assembly, possibly via an association with non-muscle myosin in Z-bodies. One alternative to this hypothesis is that contractile activity itself, absent in paralyzed *unc45b* mutants, is required for timely Z-disc maturation.

Here, we use small-molecule inhibitors of contraction to demonstrate that Z-disc maturation and organization of thick filaments into bundles require muscular contractility *in vivo* during *X. tropicalis* development. Despite the sequential posterior addition of somites, α-actinin-stained Z-discs first appear broadly in all but the youngest somites at the 11-somite stage, coinciding with initiation of a twitch response to external stimuli (Nieuwkoop and Faber, 1967). Thick filaments organized into bundles are also present at NF24. Subsequent Z-disc assembly always occurs in the second or third youngest posterior SIII/SII somite throughout the remaining tailbud stages. When muscle contraction is prevented using two different small-molecule inhibitors, only poorly organized thick filaments form, and Z-disc maturation is significantly delayed compared with controls.

## RESULTS

**Figure 1:**
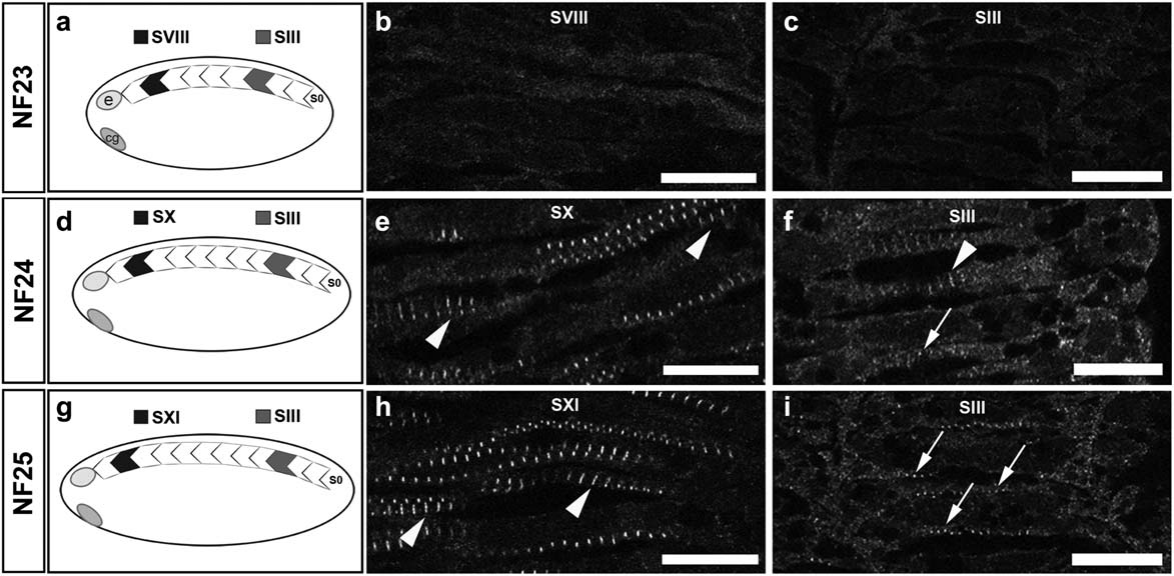
α-Actinin-stained Z-discs first appear in 11 somite embryos (a, d, g) Schema of somites imaged and somite numbering. Somites are sequentially added and numbered from the posterior during development (right, S0); second-to-anterior shaded black; third-youngest (SIII) gray; e = eye; cg = cement gland. α-Actinin staining in second-to-anterior somite in (b) nine somite NF23 (somite SVIII), (e) 11 somite NF24 (SX), and (h) 12 somite NF25 (SXI) embryos or third from posterior (SIII) somite at corresponding stage (c, f, i). Z-discs (arrowheads) are present in anterior somites from NF24 (e), becoming more abundant at NF25 (h). Z-bodies (arrows) are observed in the SIII somite of 11 and 12 somite embryos (f, i). Initial α-actinin staining appears in 11 somite embryos in both anterior and posterior somites Scale bar = 13 μm.

The delay in Z-disc maturation observed in genetically paralyzed *dit* embryos (Geach and Zimmerman, 2010) could indicate direct participation of unc45b protein in Z-disc formation (Myhre and Pilgrim, 2010), or could point to a requirement for muscle contraction in timely Z-disc assembly and sarcomere maturation. In order to test these hypotheses, we first determined when these structures initially assemble in the embryo, using established somite numbering conventions (schema in Fig. 1a, d, g) (Pourquie and Tam, 2001). Because the most anterior somite is less accessible, the second-to-anterior somite and the SIII third-from-posterior somite were imaged by confocal microscopy at NF23 (nine somites; *n* = 16), NF24 (11 somites; *n* = 13), and NF25 (12 somites; *n* = 6) for α-actinin, a key structural component of Z-discs. No Z-discs were observed in either anterior or posterior somites at NF23 (Fig. 1b, c). At NF24, α-actinin-stained Z-discs were clearly present in both anterior (Fig. 1e, arrowheads) and SIII posterior somites (Fig. 1f, arrowhead), although in posterior somites Z-discs were less frequent and punctate immature Z-bodies were present (Fig. 1f, arrow). By NF25, anterior Z-discs had become broader and more defined (Fig. 1h, arrowheads), with Z-bodies still seen in the new SIII somite (Fig. 1i, arrows). Thereafter, developing Z-discs were consistently observed in SIII throughout remaining stages of development, with Z-bodies often seen in somite SII (Fig. 4a, d, insets).

**Figure 2:**
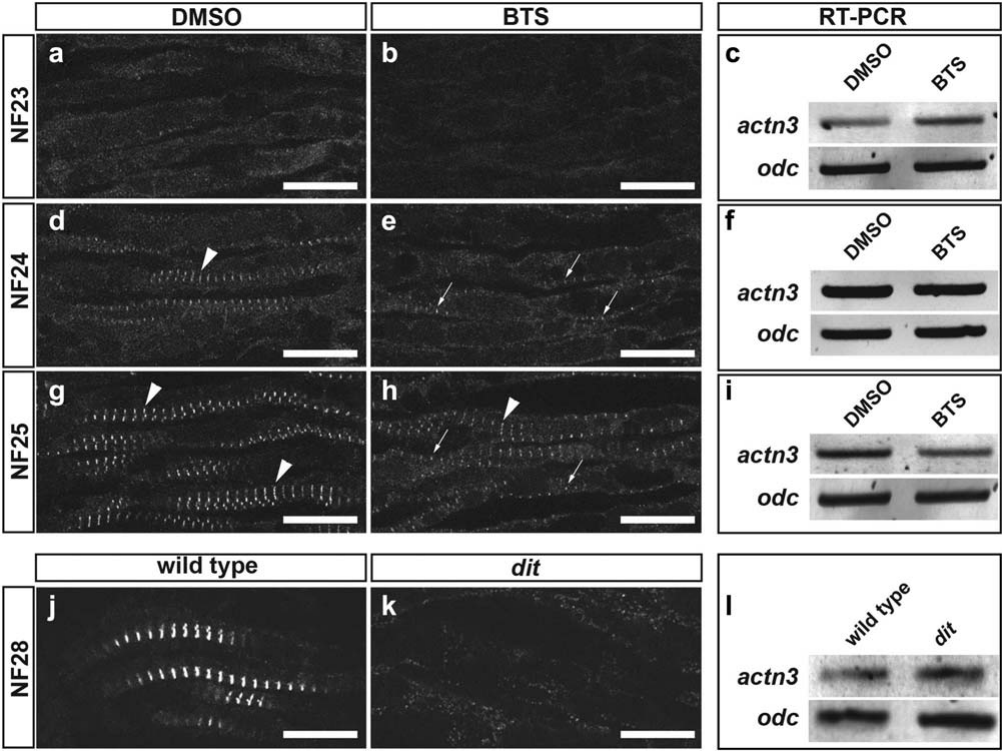
Z-disc maturation is delayed in the absence of contractility. α-Actinin staining in an anterior somite of (a, b) nine somite NF23, (d, e) 11 somite NF24, or (g, h) 12 somite NF25 embryos treated with vehicle control (a, d, g) or BTS (b, e, h) showing Z-discs (arrowheads) and Z-bodies (arrows). Z-disc maturation is delayed in BTS-treated embryos. (k) α-Actinin maturation is likewise delayed in genetically paralyzed *dit* mutant embryos at NF28 relative to (j) wild-type control. (c, f, i, l) Reverse transcription-PCR shows that *actn3* mRNA is present before Z-discs are detected and remains expressed when embryos are paralyzed by BTS treatment or the *dit* mutation. Scale bar = 18 μm.

To determine whether contractile activity is required for onset of Z-disc assembly, we inhibited muscle contraction using 1 mM *N*-benzyl-*p*-toluene sulfonamide (BTS). BTS is a specific inhibitor of skeletal muscle myosin II (Cheung *et al*., 2002), which weakens myosin ATPase activity by preventing phosphate release and decreasing myosin-ADP affinity for actin (Shaw *et al*., 2003). Embryos were cultured from NF22 (prior to muscle twitching and Z-disc assembly) and fixed for confocal microscopy at NF23, NF24, and NF25 or processed for reverse transcription-polymerase chain reaction (PCR) analysis of gene expression. As expected, no Z-discs were present at NF23 (*n* = 4, Fig. 2a, b). In BTS-treated NF24 embryos, Z-discs were infrequent and smaller than that in controls (*n* = 11, compare Fig. 2d arrowheads and 2e arrows). By NF25, Z-discs (Fig. 2h, arrowheads) were beginning to form in BTS-treated embryos (*n* = 10), but many Z-bodies remained (arrows). We then determined whether absence of contractile activity affects α-actinin transcription. Two α-actinin species (*actn2* and *actn3*) are expressed in adult skeletal muscle, but *actn2* transcripts are not present in early tailbud embryos (data not shown), consistent with their absence in EST profiles prior to tadpole stages (Unigene ID: Str.24543). *actn3* mRNA was detected in BTS-treated embryos at all stages (Fig. 2c, f, i). Z-disc formation was similarly disrupted in *dit* embryos at NF28, the earliest stage at which the mutant phenotype can be scored reliably (Fig. 2j, k), but recovered at later developmental stages (Geach and Zimmerman, 2010). As in BTS-treated embryos, *actn3* mRNA was also detected in *dit* embryos, demonstrating that the activity-dependent delayed maturation of α-actinin immunoreactivity occurred post-transcriptionally.

**Figure 3:**
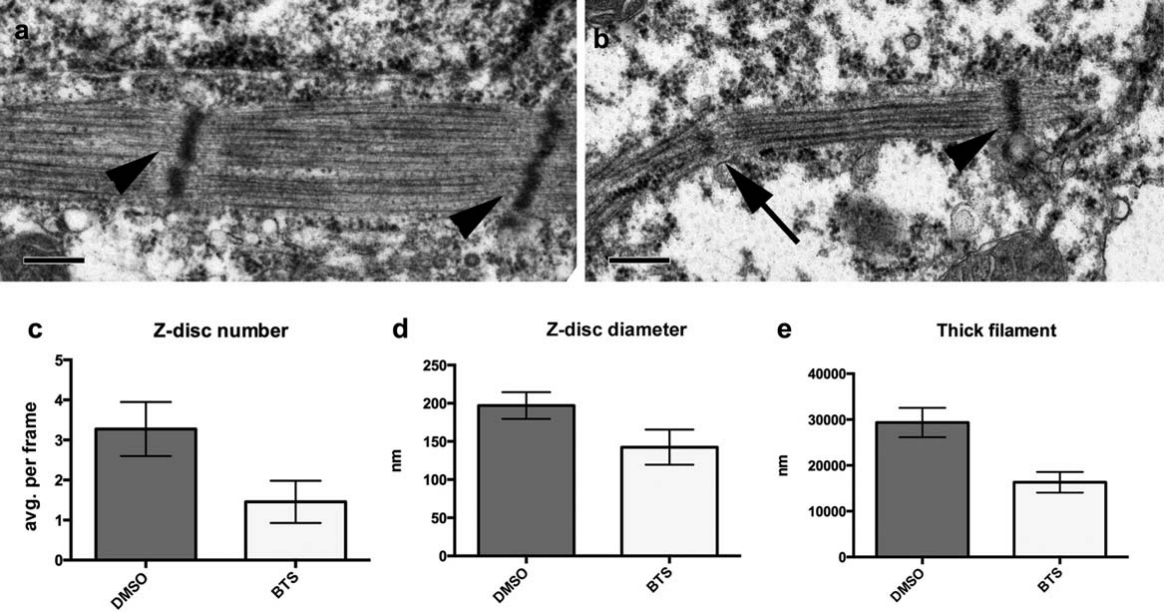
Z-disc diameter and thick filament bundling are delayed in the absence of contractility. Representative fields of TEM sections from (a) DMSO vehicle control showing well-formed Z-discs (arrowheads) and (b) BTS-treated somites with an immature Z-disc (arrow) at NF24; scale bar = 200 nm. Z-discs were identified as electron-dense regions crossing thick filaments (arrowheads). The number of Z-discs was reduced in BTS-treated embryos (c), as was their diameter (d). Thick filaments also appeared more disordered in BTS-treated embryos, and (e) average length of visible thick filaments per frame was reduced relative to controls.

**Figure 4:**
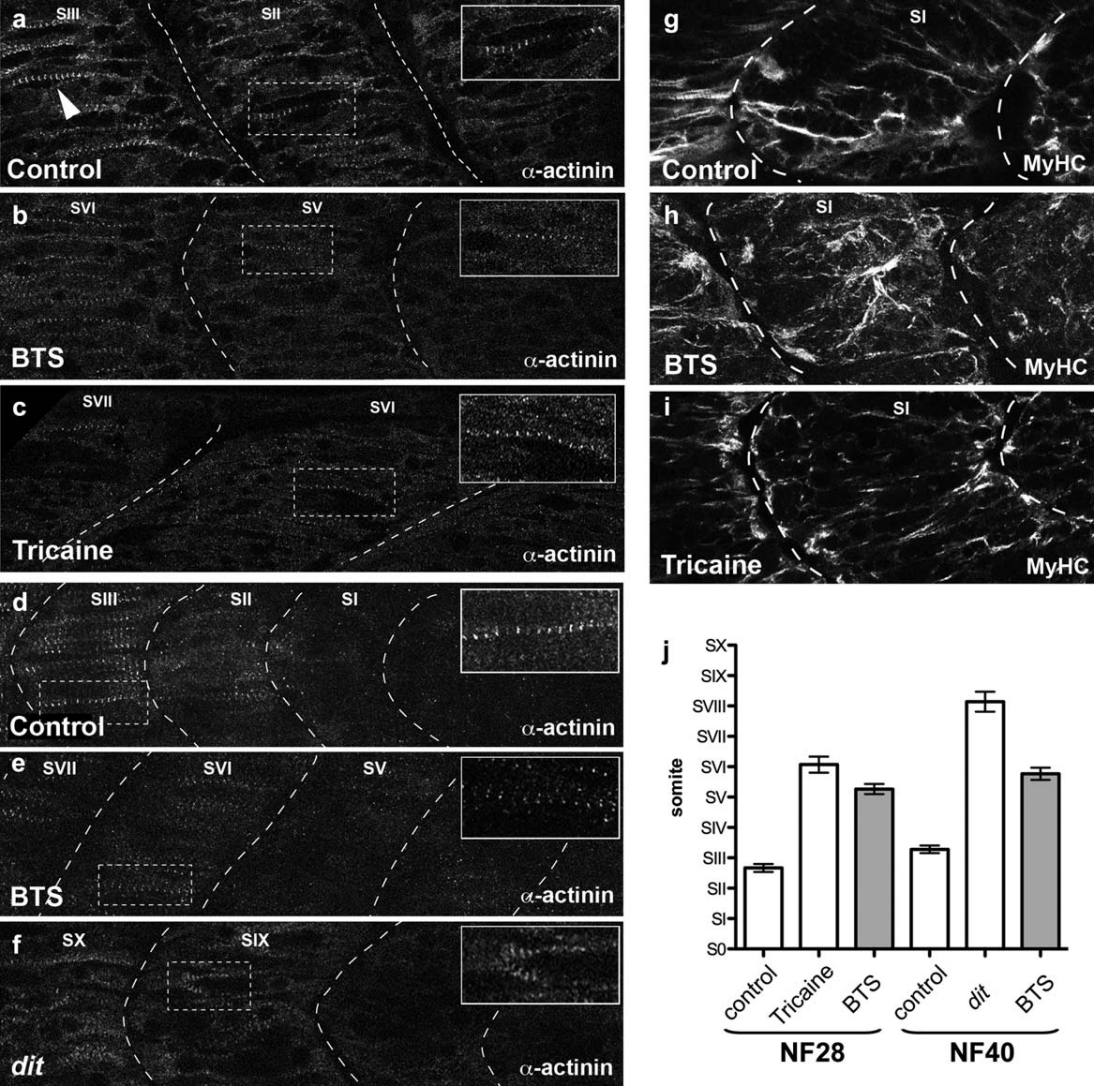
Timely Z-disc maturation in sequentially added posterior somites also requires muscle activity. At later stages, Z-disc maturation accompanies sequential addition of posterior somites, but shows consistent delay in paralyzed embryos. NF28 α-actinin-stained posterior somites treated with (a) vehicle control, (b) BTS, or (c) tricaine; intersomitic boundaries indicated by dashed lines. Z-bodies in control embryos appear in the SII somite (a, inset), with Z-discs visible in SIII (arrowhead). Paralysis with BTS or tricaine shifts Z-disc maturation to older SV–SVI somites (b, c inset). (d–f) NF40 posterior somites stained for α-actinin, showing Z-bodies in the SIII somite in control (d, inset), in the SVI somite of BTS-treated embryos (e, inset), or SIX somite in *dit* mutants (f, inset). (g–i) MyHC-stained thick filaments are unaffected in NF28 BTS- and tricaine-treated embryos. (j) Quantitation of anterior shift of the last Z-disc-containing somite at NF28 and NF40 caused by pharmacological or genetic paralysis. The youngest (most posterior) Z-disc-containing somite is shifted anteriorly by pharmacological or genetic paralysis, as shown by quantitation of anterior shift at stages NF28 and NF40 in tricaine- and BTS-treated embryos and *dit* mutants.

We also compared sarcomeric ultrastructure in normal and paralyzed NF24 somites using transmission electron microscopy (TEM). Embryos were treated with either vehicle control or BTS from NF22, fixed, processed for TEM at NF24, and imaged. Eleven image fields containing recognizable sarcomeric structures were scored for the sum of the length of individual thick filaments (total visible thick filament length), and both number and diameter of electron-dense Z-disc-like structures crossing thick filaments (representative fields shown in Fig. 3a, b). The average number of Z-discs per frame was reduced in BTS-treated embryos (1.45) compared with that in controls (3.27, *P* < 0.1; Fig. 3c), confirming our earlier results. In control embryos, clearly defined Z-discs spanned coherent bundles of thick filaments (Fig. 3a, arrowheads). In BTS-treated samples, smaller electron-dense regions consistent with immature Z-discs were seen (Fig. 3b, arrow). Average Z-disc diameter (perpendicular to thick filaments) was reduced (142 vs. 197 nm; *P* < 0.1; Fig. 3d), with no significant change in Z-disc width (data not shown). Despite little effect of paralysis on myosin heavy chain (MyHC) immunohistochemistry (Fig. 4g–i), ultrastructure showed significant reduction in total visible thick filament length compared with controls (average 16,309 vs. 29,341 nm per frame; *P* < 0.01; Fig. 3a, b, e). However, paralysis also seemed to disrupt organization of thick filaments into straight parallel bundles, possibly as a consequence of delayed Z-disc maturation. Because our analysis focuses on sarcomeric structures visible in the plane of section, the reduction in total thick filament length could be an artifact of disorganized thick filaments going out of frame more than controls.

To quantify the delay in Z-disc maturation observed in both genetically and chemically paralyzed embryos, we examined *de novo* assembly in sequentially forming posterior somites. Because the temporal rate of development varies among embryos receiving different treatments and between different genetic backgrounds, we quantified the induced delay by scoring the youngest somites with Z-discs. Embryos were cultured from NF22 for 8 h (until ∼ NF28) in either BTS or another paralyzing agent, 0.04% tricaine. Tricaine inhibits muscle action potentials, likely by blocking voltage-gated Na^+^ channels (Hedrick and Winmill, 2003). Z-discs in untreated embryos were clearly visible in the SIII somite (*n* = 21, Fig. 4a, arrowheads), with Z-bodies seen in the SII somite (Fig. 4a, inset). However, Z-discs in embryos treated with either BTS or tricaine were located in older, more anterior somites compared with controls: somite SVI for BTS-treated embryos (*n* = 19, Fig. 4b, inset) and somite SVII in tricaine-treated embryos (*n* = 29, Fig. 4c). Z-bodies were observed in the SV and SVI somites of BTS and tricaine embryos, respectively (Fig. 4b, c, insets); however, more posterior, younger somites were devoid of these structures. MyHC staining was unaffected in either BTS- or tricaine-treated embryos (Fig. 4g–i), with strong filamentous MyHC immunoreactivity in the most posterior somites (SI), further demonstrating that myosin filaments can assemble in the absence of Z-discs.

In *dit* mutant embryos at NF28, Z-discs are absent (Fig. 2k), but recover by tadpole stages (Geach and Zimmerman, 2010). We, therefore, analyzed Z-disc assembly at NF40 when Z-discs are present in *dit*, and the rate of maturation can be scored. *dit* embryos were separated from wild-type siblings at NF28 and cultured to NF40. We also treated unrelated wild-type embryos with BTS from NF22 to NF40 to assess Z-disc maturation delay at later stages. Z-discs developed as expected in the SIII somite of wild type embryos (Fig. 4d, inset). In BTS-treated embryos, no Z-disc-like structures were found posterior to approximately somite SV (*n* = 13; average SVI) (Fig. 4e, inset), consistent with results in NF28 embryos. In *dit* embryos at NF40, no Z-discs were detected posterior to somite SVIII (*n* = 14; average SIX; Fig. 4f, inset). The anterior shift in Z-disc maturation caused by either genetic or drug-induced paralysis was highly significant at both early and later stages (*P* < 0.01, Fig. 4j).

## DISCUSSION

We previously observed a delay in Z-disc maturation in the *dicky ticker* mutation (Geach and Zimmerman, 2010). Here, we asked whether this delay resulted from a direct requirement in the maturing Z-disc for the myosin co-chaperone *unc45b*, or indicated a role for myosin contraction in Z-disc and/or general sarcomere assembly.

To evaluate these hypotheses, we first characterized the onset of sarcomerogenesis in *X. tropicalis*. In zebrafish and *X. laevis*, somites develop muscle structure within the first 24 h of development (Costa *et al*., 2002; Grimaldi *et al*., 2004). The intermediate filament desmin is the first sarcomeric protein detected in both rat and zebrafish (Costa *et al*., 2002; Furst *et al*., 1989), but, in our hands, available desmin antibodies did not stain *X. tropicalis* reliably. The first molecular indicator of sarcomerogenesis we reproducibly observed was small α-actinin-stained Z-discs at NF24. Fast muscle-specific MyHC staining has been detected in *X. laevis* slightly earlier at NF22 (Grimaldi *et al*., 2004), but the difference could be due to internal versus external stage criteria (used in Grimaldi *et al*., 2004) or a species difference.

Given the strict craniocaudal progression of somite differentiation (Pourquie, 2011), we expected anterior somites to undergo sarcomerogenesis first, gradually followed by posterior somites. However, this was not observed in the initial phase of sarcomere assembly. Z-discs were first seen in 11 somite embryos (NF24 in *X. tropicalis*) in both anterior and posterior regions, coinciding with onset of a muscular twitch response to stimulation. At subsequent stages, Z-discs were always observed in the SIII somite (occasionally SII) in sync with the addition of new somites from the presomitic mesoderm. Because Z-discs, and their coupling to costameres, are required for force transmission during muscular contraction, it might be assumed that contractile activity would not precede Z-disc assembly. However, our results indicate that early stages of Z-disc maturation and/or thick filament bundling require acto-myosin activity. Interestingly, expression of α-actinin mRNA was detected in NF22 (eight somite) embryos despite absence of α-actinin immunoreactivity at this stage. The absence of α-actinin-stained Z-discs, despite expression of *actn3* mRNA, suggests that post-transcriptional or -translational mechanisms may help regulate maturation. Indeed, sarcomeric proteins, including α-actinin, can be detected on Western blots of chicken embryonic hearts before individual sarcomeres appear (Ehler *et al*., 1999). Unfortunately, available α-actinin antibodies do not work in Western blots with *Xenopus* sp., but our ultrastructure analysis suggests that some immature Z-body-like structures are present in paralyzed embryos.

Previous studies of cultured myocytes treated with inhibitors of acto-myosin interactions or calcium release have noted gross defects in myofibril assembly (thinner, branched myofibrils), with no change in sarcomere number, consistent with a requirement for muscle contractility in myofibril assembly (Ferrari *et al*., [Bibr b14],[Bibr b13]; Ramachandran *et al*., 2003; Soeno *et al*., 1999). However, no assessment was made of Z-disc or A-band assembly. More recently, two groups have shown that paralysis during zebrafish embryogenesis reduces myofibril assembly and translation of myofibril-specific proteins (Etard *et al*., 2007; Yogev *et al*., 2013). In our hands, Z-disc maturation is retarded in the absence of contraction at both early and late tailbud stages. Moreover, our results suggest that although thick filaments assemble on schedule, their organization into appropriately sized bundles is disrupted, possibly as a consequence of Z-disc absence.

During sarcomerogenesis, Z-discs align on actin filaments, but how they do so remains under debate. In one model, NMMII “premyofibrils” align actin filaments with α-actinin-rich Z-bodies, which fuse with their neighbors to form Z-discs, as sarcomeric MyHC gradually replaces NMMII in the sarcomere (Dabiri *et al*., 1997; Du *et al*., 2003; Sanger and Sanger, 2008; Sanger *et al*., 2005). However, the premyofibril model does not incorporate a contribution from contraction. Using cultured chick cardiomyocytes transfected with an α-actinin-GFP fusion construct, Dabiri *et al*. (1997) observed Z-bodies developing on premyofibrils and maturing into Z-discs. α-Actinin initially aligned on the premyofibrils as punctate staining, as sarcomerogenesis progressed Z-bodies on neighboring fibers aligned and fused to form Z-discs in the mature sarcomere. Recent modeling studies (Friedrich *et al*., 2011) predict that contractility and elastic interactions with the ECM can organize and bring into register Z-bodies on neighboring fibers to facilitate Z-disc maturation (Dabiri *et al*., 1997). Our data seem to support such a mechanism. Z-disc assembly is delayed in chemically or genetically paralyzed embryos, consistent with a failure to correctly align neighboring Z-bodies. Moreover, our ultrastructure analysis shows smaller electron-dense structures consistent with formation of Z-bodies in paralyzed sarcomeres, but a delay in lateral alignment of these to form mature Z-discs.

Z-discs themselves contribute to the transmission of contractile force in muscle. In *Drosophila*, the appearance of Z-discs correlates with an increase in contraction throughout the whole embryo (Katzemich *et al*., 2013). Interestingly, assembly of costameres, which link Z-discs to the sarcolemma and are the main site of contractile force transmission, is also thought to require contractility (Danowski *et al*., 1992; Sharp *et al*., 1997; Simpson *et al*., 1996). Given that Z-discs eventually form even in fully paralyzed embryos, a contraction-independent mechanism might also function. Myhre and Pilgrim (2012) hypothesize that unc45b might also regulate directly Z-disc assembly, possibly via NMMII. However, we observe similar defects in Z-disc assembly in both the *dit* mutation and by paralysis with two small-molecule inhibitors, suggesting that contraction contributes significantly to α-actinin nucleation, and that defective unc45b function cannot fully account for the *dit* Z-disc phenotype. Furthermore, BTS is specific for skeletal muscle myosin, having at least 100 times less affinity for NMMII; so, it is unlikely that its effects are being mediated by non-muscle myosin. It remains possible that timely Z-disc assembly requires both contractility and a direct role for unc45b in NMM maturation. We have been unable to investigate the latter hypothesis *in vivo* because of toxicity of NMM-targeted compounds, such as blebbistatin, to *X. tropicalis*. However, our results show that blocking muscle contraction *in vivo* using either of two different small-molecule inhibitors or mutation of the myosin chaperone *unc45b* produces similar deficits in Z-disc assembly, highlighting an important role for contractility in surprisingly early steps in sarcomerogenesis.

## MATERIALS AND METHODS

### Breeding

Wild-type *X. tropicalis* embryos were generated from outbred laboratory stocks. *dit* mutants were obtained by conventional mating of carriers or gynogenesis (Goda *et al*., 2006).

### Embryo Staging

External criteria defined for *X. laevis* (Nieuwkoop and Faber, 1967) were used for initial staging. Somites were counted in cleared embryos after antibody staining. Somite number was found to differ from *X. laevis* at corresponding external criteria stages, with nine to 10 somites at NF23 (12 in *X. laevis*), 11 somites at NF24 (15 in *X. laevis*), and 12 somites at NF25 (16 in *X. laevis*). Somites were numbered from the youngest posterior somite according to Pourquie and Tam (2001).

### Immunohistochemistry

Embryos were fixed in 4% paraformaldehyde and stained with either α-MyHC (A4.1025. DSHB, University of Iowa, Iowa City, IA) or α-actinin (EA-53, AbCam, Cambridge, UK), with Alexa-conjugated secondary antibodies (Invitrogen, UK). Pre-NF30 embryos were dehydrated in methanol and cleared in benzyl alcohol:benzyl benzoate (2:1). Images were obtained on a Zeiss AxioImager 2 confocal microscope and analyzed using Volocity (PerkinElmer) and ImageJ (NIH).

### Electron Microscopy

Samples were fixed in 2% glutaraldehyde, 2% paraformaldehyde followed by 1% osmium tetroxide in 0.1 M sodium cacodylate buffer pH 7.2, then aqueous uranyl acetate and dehydration through a graded ethanol series and propylene oxide. Samples were embedded in Epon, and 50-nm sections were mounted on pioloform-coated grids and stained with ethanolic uranyl acetate followed by Reynold’s lead citrate. Sections were viewed with a Jeol 100EX and photographed using a Gatan Orius 1000CCD. Thick filament and Z-discs were measured in ImageJ. Briefly, the line tool was used to measure every visible thick filament per frame. Z-discs were identified as electron-dense regions crossing thick filaments. Z-disc diameter (perpendicular to thick filament) and width (parallel to thick filament) were also measured using the line tool. Statistical significance was determined using *t*-tests.

### Paralysis Assays

Embryos were incubated at room temperature in 0.1% dimethyl sulfoxide (DMSO) vehicle control, 0.04% tricaine, or 1 mM BTS (1 M stock in DMSO) (Sigma-Aldrich, UK) in 1/20^th^ MMR with gentamycin, cultured to the appropriate stage and processed for immunohistochemistry. Paralysis was confirmed by hourly probing with forceps. Statistical significance was determined using Student’s *t*-test.

### Reverse Transcription-PCR

RNA was extracted with Trizol (Invitrogen) from pools of five embryos. cDNA was synthesized using Superscript III RT (Invitrogen) and random hexamers. Semi-quantitative PCR was performed using Taq polymerase (NEB) under the following conditions: 92 °C for 2 min, (92 °C for 30 s, 56 °C for 30 s, and 72 °C for 1 min, for the indicated number of cycles) and 72 °C for 5 min, with the following primers: *α-actinin-3* (*actn3*) forward: ggagcttgcccgtcagcagg; reverse: ccatgctgtagtttgtgtgc (27 cycles) and *ornithine decarboxylase* (*odc*) forward: gccagtaagacggaaatcca; reverse: cccatgtcaaagacacatcg (26 cycles).

### Ethics Statement

All animal use complied with the UK Animals (Scientific Procedures) Act 1986 under Home Office Project Licence PPL 80/2294. Ethical review of the project and all regulated procedures were carried out by the National Institute for Medical Research Ethical Review Panel. Procedures involving regulated organisms were limited to induction of ovulation/breeding and terminal anesthesia using approved methods.
